# Fabrication and Evaluation of a Graphene Oxide-Based Capacitive Humidity Sensor †

**DOI:** 10.3390/s16030314

**Published:** 2016-03-01

**Authors:** Jinfeng Feng, Xiaoxu Kang, Qingyun Zuo, Chao Yuan, Weijun Wang, Yuhang Zhao, Limin Zhu, Hanwei Lu, Juying Chen

**Affiliations:** 1 School of Mechanical Engineering, Shanghai Jiao Tong University, Shanghai 200240, China; fengjf@sjtu.edu.cn; 2 Process Technology Department, Shanghai IC R&D Center, Shanghai 201210, China; zuoqingy@icrd.com.cn (Q.Z.); yuanchao@icrd.com.cn (C.Y.); wangweijun@icrd.com.cn (W.W.); yhzhao@icrd.com.cn (Y.Z.); 3 PIE Department III, Shanghai Huahong Grace Semiconductor Manufacturing Corporation, Shanghai 201206, China; limin.zhu@hhgrace.com (L.Z.); hanwei.lu@hhgrace.com (H.L.); juying.chen@hhgrace.com (J.C.)

**Keywords:** relative humidity, humidity sensor, graphene oxide, capacitive sensing

## Abstract

In this study, a CMOS compatible capacitive humidity sensor structure was designed and fabricated on a 200 mm CMOS BEOL Line. A top Al interconnect layer was used as an electrode with a comb/serpent structure, and graphene oxide (GO) was used as sensing material. XRD analysis was done which shows that GO sensing material has a strong and sharp (002) peak at about 10.278°, whereas graphite has (002) peak at about 26°. Device level CV and IV curves were measured in mini-environments at different relative humidity (RH) level, and saturated salt solutions were used to build these mini-environments. To evaluate the potential value of GO material in humidity sensor applications, a prototype humidity sensor was designed and fabricated by integrating the sensor with a dedicated readout ASIC and display/calibration module. Measurements in different mini-environments show that the GO-based humidity sensor has higher sensitivity, faster recovery time and good linearity performance. Compared with a standard humidity sensor, the measured RH data of our prototype humidity sensor can match well that of the standard product.

## 1. Introduction

With the developing of the Internet of Things and sensor networks, MEMS/sensor “consumerization” has begun to emerge, and more and more new applications of MEMS/sensors have begun to be found in consumer products such as mobile phones, PAD, *etc.* With the consumer market growth, requirements for high performance and low cost MEMS/sensors are increasing, especially for digital multimedia products [1,2]. Humidity sensors is some of the most widely used sensors, found in industry control, automobile defogger, agriculture, and environment monitoring applications, *etc.* Recently, humidity sensors have been successfully integrated into high-end mobile phone products including the Samsung Galaxy S4, and their market will increase dramatically with the growing consumer electronics demands.

Based on their measurement units, humidity sensors can be divided into two types: relative and absolute humidity sensors. According to the operating principles, these are three main types: Capacitive, resistive, and thermal conductivity humidity sensors. Most humidity sensors are capacitive RH sensors because of their low cost, linearity, minimal long-term drift and hysteresis [3].

Graphene is one of the most promising materials developed in this century with its exceptional mechanical, thermal and electrical properties [4,5,6,7]. GO is made up of single or several closely-spaced graphene sheets, and has sp^2^ and sp^3^ hybridized carbon atoms which can be considered as insulators [8]. GO is slightly soluble in water, and has plenty of oxygen-containing functional groups, such as epoxy, hydroxy (–OH), and carboxy (–COOH) groups, covering its surface [9,10,11,12]. GO materials have already attracted significant interest in various sensing applications because of their ultrasensitive detection properties [13,14,15,16,17], but little work has been done on evaluation of its critical properties for sensor applications, such as leakage current control, CMOS compatible performance, stability, linearity, *etc.*

In this work, a capacitive humidity sensor was designed and fabricated with GO as sensing material. The capacitance of the sensor device was increased about four times from ~22.5% RH to ~85% RH, which shows excellent sensitivity. After integrating the sensor device with a dedicated readout ASIC, a prototype humidity sensor was built and its measured data was compared with that of a standard humidity sensor.

## 2. Experimental Details

**Figure 1:**
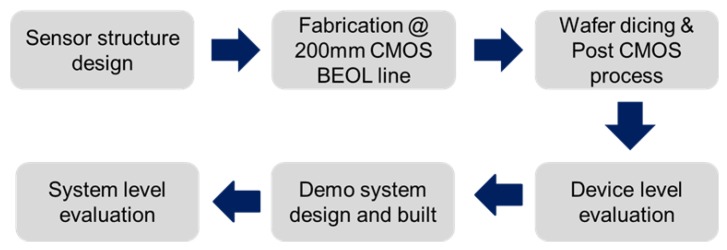
Workflow of this study.

Figure 1 shows the whole workflow of this study. Firstly, The CMOS compatible capacitive sensor structure was designed and fabricated on a 200 mm CMOS BEOL line. After that, the wafer dicing was done, and the sensor was fabricated by filling GO material in this structure. Finally the prototype humidity sensor was designed and built to evaluate its performance.

**Figure 2:**
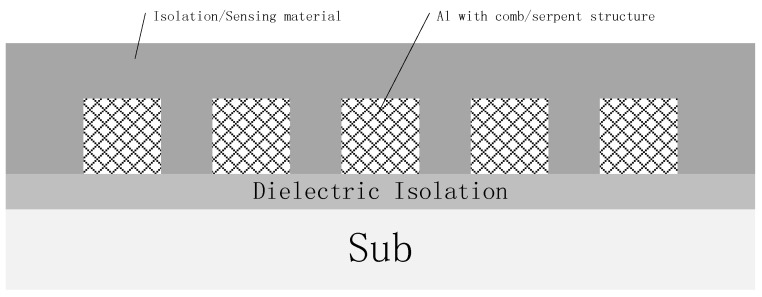
Schematic cross-sectional-view of designed humidity sensor structure.

The sensor structure was designed and fabricated with a comb/serpent capacitor structure by a standard 0.35 μm CMOS compatible process, as shown in Figure 2. The top Al interconnect layer was used as the capacitor electrode with a line/space/height = 0.78 μm/0.54 μm/0.8 μm. After the sensor structure fabrication, a full wafer CV/IV mapping test was done to check the process uniformity and leakage performance.

After wafer dicing was finished, and the sensor was fabricated by filling GO material in the structure. In this post CMOS process, a GO-based water dispersion solution was used as filling material, and after the filling process the sensor was thermally treated to remove the water. XRD was used to check the sensing material properties, and CV/IV data was measured in different environments to check the leakage and capacitance properties.

**Figure 3:**
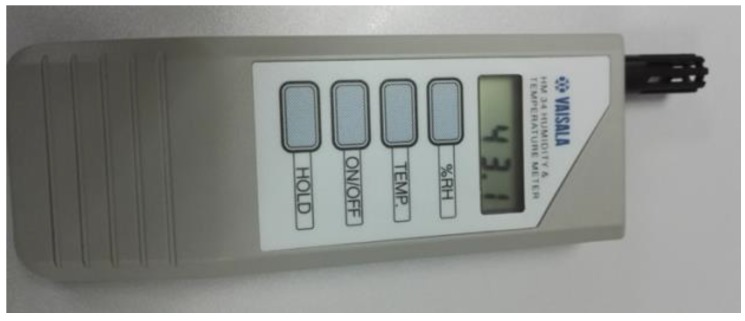
Standard temperature and humidity sensor.

A prototype humidity sensor was designed and built by integrating the sensor with a dedicated readout ASIC and display/calibration module at the PCB level. A standard frequency-voltage convertor (CAV444, Analog Microelectronics, Mainz, Germany) was used as readout circuit to extract the capacitance variation by the RC oscillation mechanism. The prototype sensor was then evaluated in different environments, and all the measured data was compared with a standard humidity sensor (HM 34 Humidity & Temperature Meter, VAISALA, Vantaa, Finland) as shown in Figure 3.

**Figure 4:**
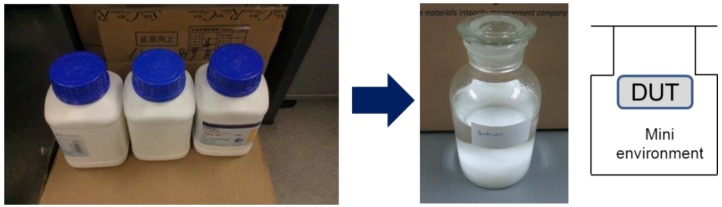
Mini-environment built with a saturated salt solution sealed in glass chamber.

All the measurements were done at a room temperature of 25 °C. CV was measured by a 4284A instrument (Agilent, Santa Clara, CA, USA) and IV was measured by an Agilent 4156C device. This study adopted a very convenient way to measure and calibrate humidity sensors by using saturated salt solutions. As shown in Figure 4, the saturated salt solution, which is made up as a slushy mixture with distilled water and chemically pure salt, is enclosed in a sealed glass chamber to build mini-environments with different RH values.

The RH of the atmosphere above the saturated salt solution in the sealed chamber was fixed at a given temperature, and it can cover almost the entire range of relative humidity, as shown in Table 1. Because the concentration of a saturated salt solution is fixed at given temperature, it is very easy to determine the saturation state when there is solid phase salt left in the solution. As the RH value will be disturbed during putting device under test (DUT) into the mini-environments, all the measurements will be done after the RH value of the mini-environment is stable which can be easily determined by using the output data of a standard humidity sensor.

## 3. Results and Discussion

**Figure 5:**
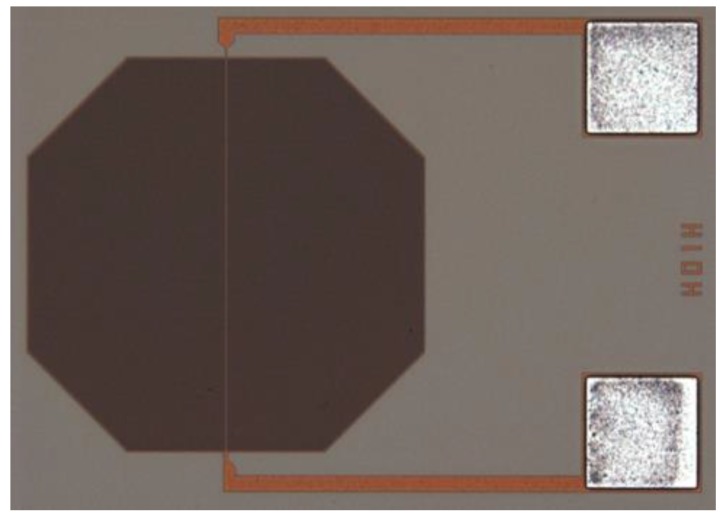
Top view photo of the fabricated sensor structure.

A standard 0.35 μm CMOS BEOL process was used to fabricate the comb/serpent capacitor structure. A BEOL Al metal layer was used as capacitor electrode with line/space/height = 0.78 μm/0.54 μm/0.8 μm. The structure was designed with consideration of least fringing field and parasitic capacitance, and PAD was formed by the same metal layer. A top-view photo of the structure can be seen in Figure 5, in which the sensor structure was located on the left, with dimensions of about 500 μm × 500 μm and the bonding PAD was on the right with dimensions of 150 μm × 150 μm.

**Figure 6:**
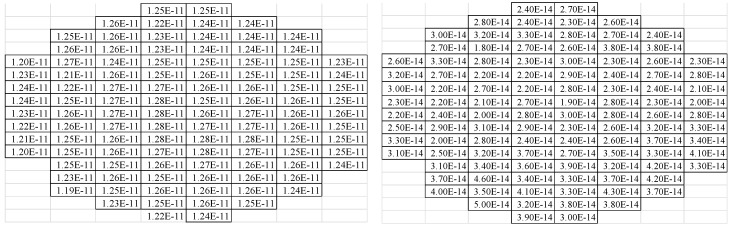
Full wafer CV (**Left**) and IV (**Right**) mapping test data of the sensor structure.

After wafer fabout, a CV and IV mapping test was done to check the uniformity and leakage performance of the structure. As can be seen in Figure 6, the capacitance of the sensor structure has a mean value of ~1.25E^−11^ f, and a very good uniformity can be obtained with a standard deviation of ~1.76E^−13^ f and NU% of ~ 1.41%. From the IV data, the structure shows a very good leakage control with a max leakage current of ~5E^−14^ A.

**Figure 7:**
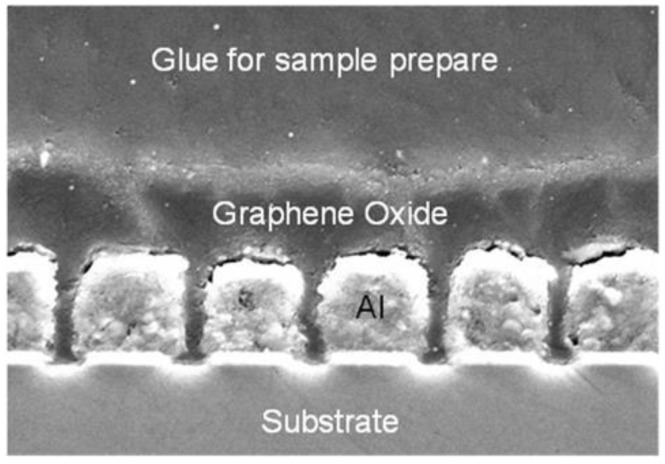
X-SEM photo of the sensor device after GO filling.

After wafer dicing, the sensor was fabricated by filling GO material in the sensor structure. A GO-based water dispersion solution was used as filling material, and after the filling process the sensor was treated in a baking oven at a temperature of 60 °C for about 50 min to remove the water. Cross-sectional SEM was done to check the filling performance, and the GO filling performance can meet the sensor requirements as can be seen in Figure 7.

**Figure 8:**
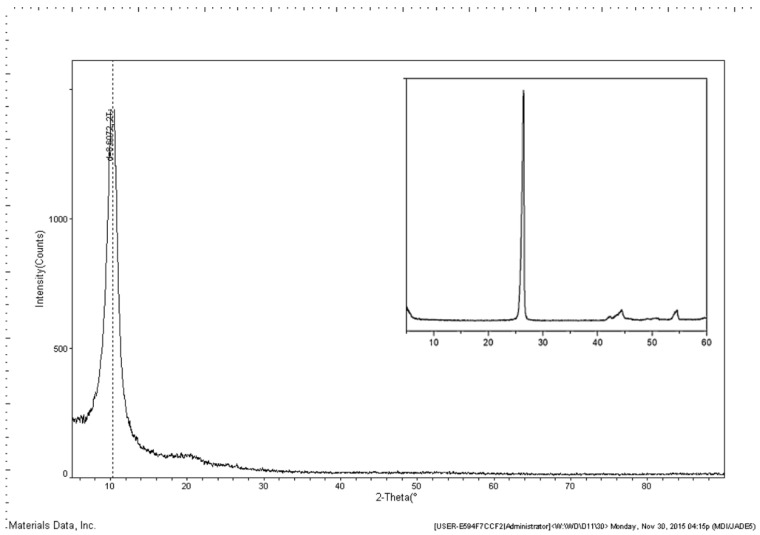
XRD photo of the GO sensing material.

GO film was prepared on a blanket substrate to do XRD analysis. Compared with graphite, GO has a variety of reactive oxygen groups, such as carboxylic acids (COOH), carbonyls (C=O), epoxides (–O–) and hydroxyls (–OH), which are linked to a C-sp^3^ structure network. Because of the existence of oxygen functional groups and some other structural defects, the interlayer distance of GO is greatly increased [12,13,14,15]. As can be seen in Figure 8, the XRD pattern of GO shows a strong and sharp (0 0 2) peak at about 10.278°, which corresponds to an interlayer distance of about 8.606 Å. The insert is the XRD pattern of graphite, which shows the (0 0 2) peak at about 26.56° corresponding to an interlayer distance of 3.34 Å.

**Figure 9:**
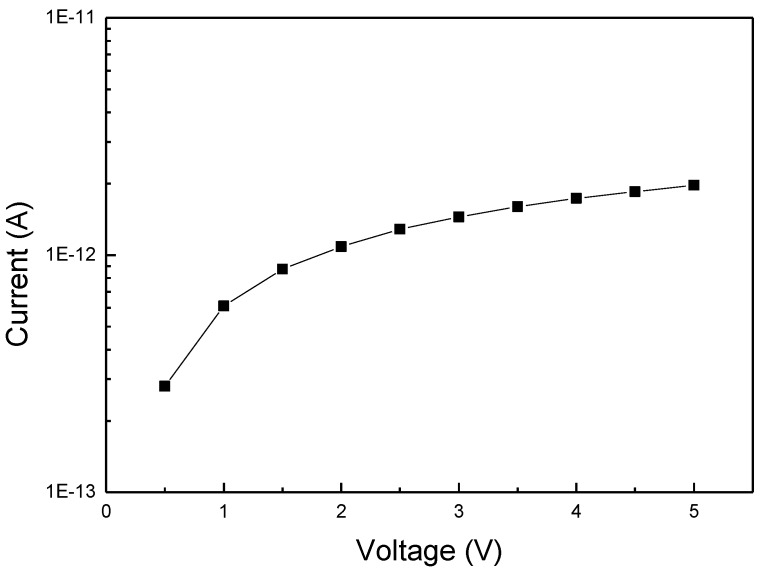
IV curve of the sensor device after GO filling.

Leakage is very critical to any sensor application with a capacitive sensing mechanism, because more leakage current will make capacitance measurement inaccurate and cause the device to malfunction. Leakage of the sensor device was measured in voltage sweeping mode from 0 V to 5 V. As shown in Figure 9, the sensor leakage can be controlled below 1E^−11^ A with a comb/serpent electrode length of ~165 mm at about 50% RH level, which can meet well the sensor application requirements.

**Figure 10:**
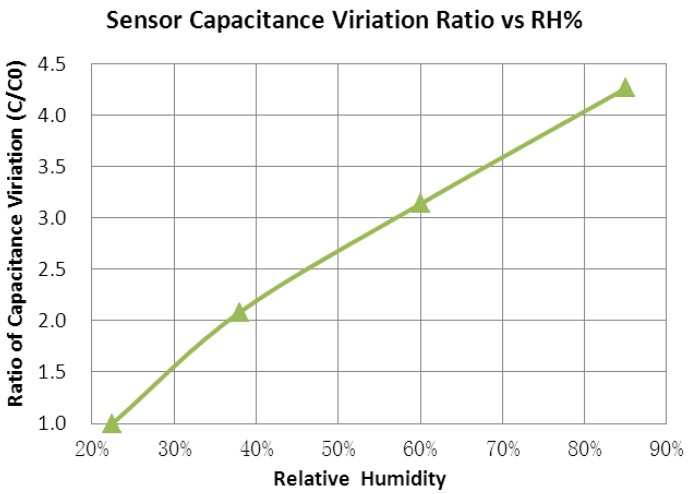
Sensor capacitance variation ratio of C/CO with different RH.

Capacitance was measured at frequency of 100 kHz by an Agilent 4284A instrument. C/C0 ratio is plotted in Figure 10, in which C is the capacitance at given RH level and C0 is the capacitance at minimal RH level of ~22.5% RH. With the RH value changing from ~22.5% to ~85%, the sensor capacitance has increased around four times, whereas normally used humidity materials, such as polyimide, only have a capacitance variation of about 20%. In addition to plenty of oxygen functional groups on the GO material, its high surface to volume ratio also contributes to this larger capacitance change.

**Figure 11:**
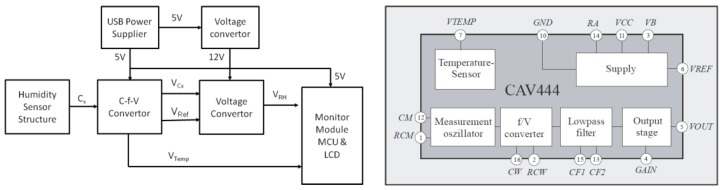
Schematic circuit block of the prototype sensor (**Left**) and block diagram of the CAV444 (**Right**).

To evaluate the potential value of GO material in humidity sensor applications, a prototype humidity sensor was designed and built by integrating the sensor with a dedicated readout ASIC and a display/calibration module on the PCB level. A standard Analog Microelectronics CAV444 frequency-voltage convertor was used as a readout circuit to extract the capacitance variation caused by the RC oscillation mechanism. A schematic circuit block of the prototype sensor and block diagram of the frequency-voltage convertor are shown in Figure 11.

**Figure 12:**
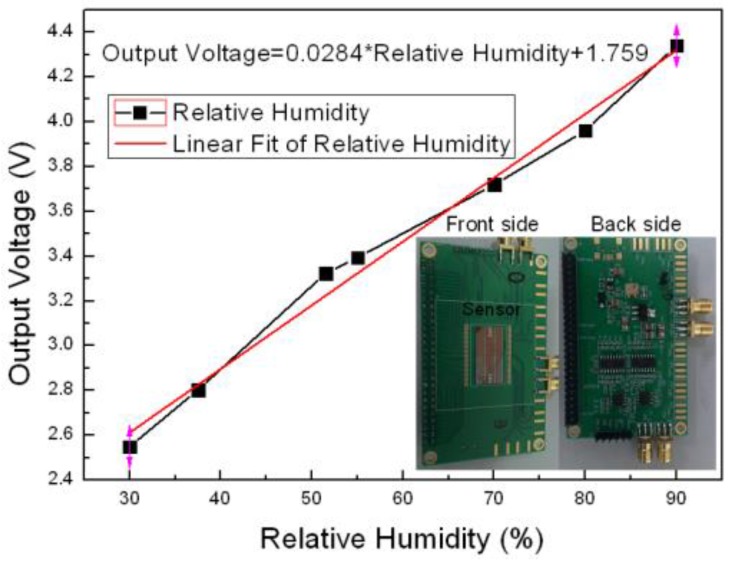
Output voltage *vs* RH value of the sensor structure.

To build the prototype sensor, the sensor device was firstly wire-bonded with the CAV444 on the PCB level, which can convert the change of capacitance to voltage. As can be seen in Figure 12, the output voltage has a very good linear relation with the RH value, and the insert graph shows the PCB photo after integrating the sensor device with the ASIC.

**Figure 13:**
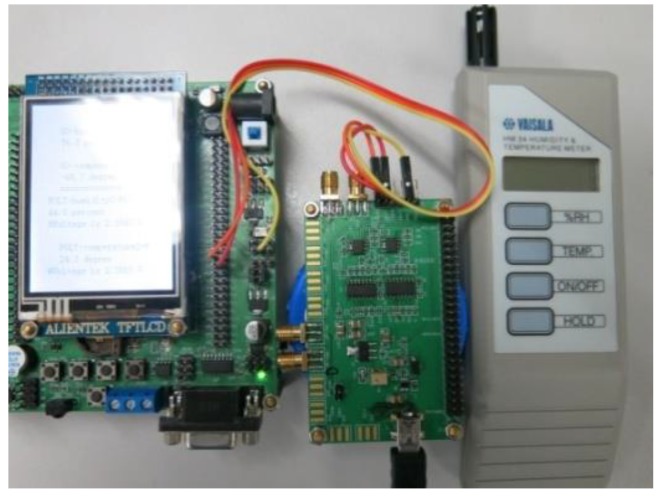
Prototype sensor with LCD display module (**Left**), PCB integration of sensor device and ASIC (**Middle**) and standard humidity sensor (**Right**).

After that, the prototype humidity sensor was built by further integrating a LCD display and calibration module, which can be seen in Figure 13.

**Figure 14:**
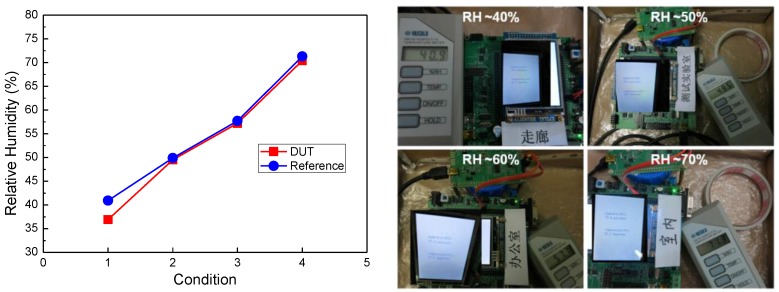
Measured humidity data of the prototype sensor (**Left**) and measurement photo of the prototype sensor and standard sensor (**Right**).

After linear calibration, the prototype sensor was used to measure the RH value in environments at different RH levels. The measured data was then compared with that of a standard humidity sensor, as shown in Figure 14. It can be seen that output humidity data of our prototype sensor can match well the output of the standard sensor.

**Figure 15:**
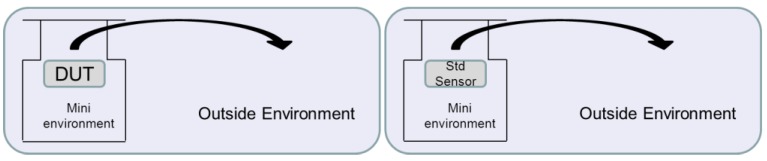
Schematic recovery time test procedure.

Response/recovery time was defined as the time (τ) required to achieve ~63% value of a humidity step function. Recovery time was measured by a simple method as shown in Figure 15. The standard and prototype sensor was put into a mini-environment of the same saturated salt solution. After stabilization, the sensor was quickly taken out from the high RH mini-environment to the outside atmosphere, and the recovery time was measured. From ~93% RH (mini-environment) to ~52% RH (outside environment), the recovery time of this work is less than 3 s compared with ~8 s for the standard humidity sensor, so the GO-based humidity sensor shows about two times faster recovery time than the standard sensor, which is due to its special structure with much higher surface to volume ratio.

## 4. Conclusions

In this work, a CMOS compatible capacitive humidity sensor structure was designed and fabricated on a 200 mm CMOS BEOL Line. BEOL Al with a comb/serpent structure was used as electrode, and GO was used as sensing material. The sensor device shows excellent sensitivity and faster recovery time than a standard sensor. A prototype humidity sensor was designed and built by integrating the sensor device with a dedicated ASIC at the PCB level, and the output value of the prototype sensor was shown to match well that of a standard humidity sensor.

## Figures and Tables

**Table 1:** Relative humidity (RH) values of normally used saturated salt solutions.

Saturated Salt Solution	Temperature (°C)	Relative Humidity (%)
LiCl	25	15
CaCl_2_	25	31
K_2_CO_3_	25	43
NaCl	25	75
KCl	25	84
K_2_SO_4_	25	98
